# Back to the future: how human induced pluripotent stem cells will transform regenerative medicine

**DOI:** 10.1093/hmg/ddt379

**Published:** 2013-08-14

**Authors:** Clive N. Svendsen

**Affiliations:** The Regenerative Medicine Institute, Cedars-Sinai Medical Center, AHSP, 8th Floor, 8700 Beverly Boulevard, Los Angeles, CA 90048, USA

## Abstract

Based on cloning studies in mammals, all adult human cells theoretically contain DNA that is capable of creating a whole new person. Cells are maintained in their differentiated state by selectively activating some genes and silencing. The dogma until recently was that cell differentiation was largely fixed unless exposed to the environment of an activated oocyte. However, it is now possible to activate primitive pluripotent genes within adult human cells that take them back in time to a pluripotent state (termed induced pluripotent stem cells). This technology has grown at an exponential rate over the past few years, culminating in the Nobel Prize in medicine. Discussed here are recent developments in the field as they relate to regenerative medicine, with an emphasis on creating functional cells, editing their genome, autologous transplantation and how this ground-breaking field may eventually impact human aging.

## INTRODUCTION

Regenerative medicine is a new and expanding area that aims to replace lost or damaged tissues in the human body through either cellular transplantation or endogenous repair. Adult stem cells infused into the circulation are currently leading the clinical front of regenerative medicine. However, there is general acceptance that mesenchymal cells, cord blood, adipose tissues and other adult stem cell sources often do not survive for more than a few weeks in patients, and their effects are most likely through growth factor release, host inflammatory responses and vascular alterations rather than replacing tissues lost in the disease. To achieve this, it will be necessary to either grow new tissues within the affected organ, or transplant powerful cells that can integrate, survive and produce new functional tissues. Fourteen years ago, human embryonic stem cells (hESCs) were isolated from the inner cell mass of embryos and could be expanded indefinitely while retaining the potential to make any cell of the body (1) and as such represented perhaps the ideal source for exploring cell therapy and endogenous repair in humans. However, there have been major roadblocks associated with (i) ethical issues with the isolation of hESCs, (ii) appropriate differentiation to mature functional phenotypes, (iii) potential immune rejection of the cells and (iv) possible tumor formation from residual pluripotent cells.

**Figure 1.**
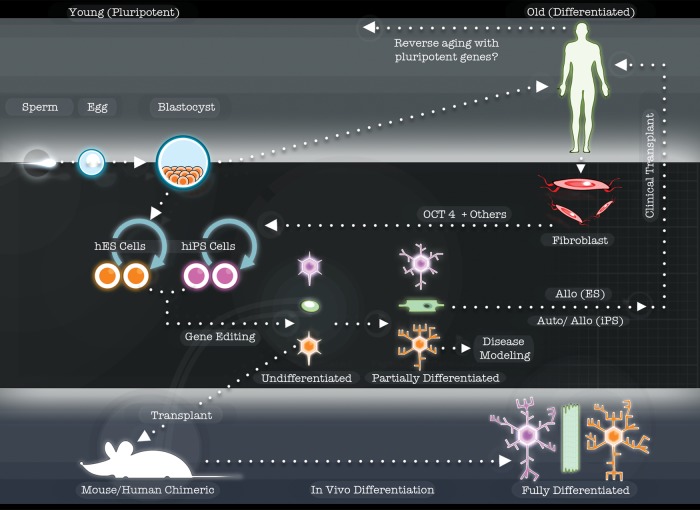
Schematic showing that the blastocyst (upper left) can either develop into a person or provide a source of hESCs (left side). Adult fibroblasts (right side) can be reprogrammed, using Oct4 and other factors, to a pluripotent state to produce hiPSCs (middle) that are similar to hESCs. Human ES and iPS cells are capable of differentiating into various immature cell types in the dish (partially differentiated), which can be used for disease modeling. In some cases, full maturation may require 3-D environments or transplantation into whole animals (bottom left). For clinical transplants, both pluripotent cell types can be used for allografts, but only hiPSCs can provide autologous grafts into patients (upper right). Finally, understanding more about how reprogramming works may allow us to reverse the aging process in humans (top arrow to left).

**Figure 2.**
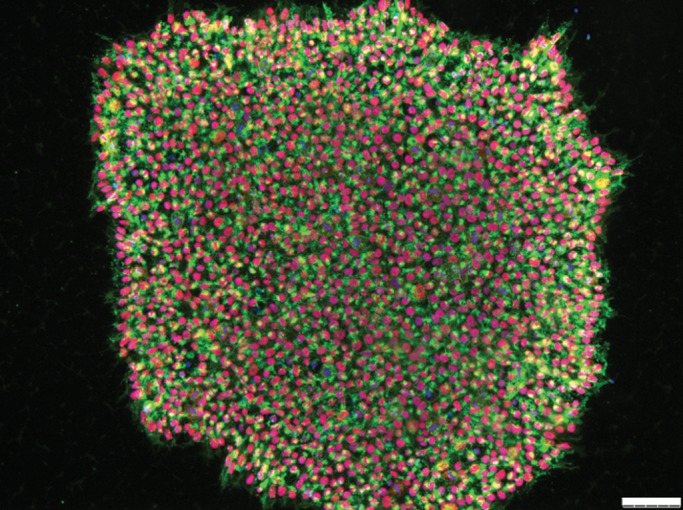
Representative human iPSC colony expressing the pluripotent markers SSEA4 (green) and Oct4 (red) shown by immunocytochemistry with nuclei stained with Dapi (blue). Scale bar 75 μm.

Recent events have moved the field to a new and exciting level of expectation. It has long been assumed that most somatic cells of the body retain the DNA required to produce a whole new organism. Indeed, somatic nuclear transfer techniques leading to cloned frogs and mammals were proof of concept that this was true (2,3). However, it was revolutionary when Takahashi and Yamanaka (4) showed in 2006 that adult mouse fibroblasts could also be sent back in time to an embryonic-like state by simply exogenously expressing powerful pluripotency transcription factors. This was followed by similar experiments in human fibroblasts a few years later (5–7) and even more recently, different sets of transcription factors have been shown to directly convert adult cells into different lineages (8). Unlike cloning techniques which have remained extremely difficult for human cells and have only been proven to work in a single very recent publication (9), reprogramming using transcription factors to produce human induced pluripotent stem cells (hiPSCs) is simple, reliable and yields a very usable cell type that is in most aspects similar to hESCs (Figs 1 and 2).

Clearly, using hiPSCs in regenerative medicine removes the hESC-associated ethical issues that resulted in restricted funding of this research in the USA and other countries. It also raises the possibility of autologous transplantation. However, other challenges remain similar to those faced by the hESC field, such as appropriate differentiation of the cells and the risk of tumor formation following grafting. In addition, there is active discussion about whether hiPSCs may be more unstable than hESCs due to their forced reprogramming, although earlier concerns regarding integration of reprogramming factors have been largely overcome by non-integrating techniques (10,11) and very recently a completely chemically defined process using small molecules to create iPS lines at least from mice but not yet with human cells (12).

Great excitement comes from the new field of disease modeling that is made possible by hiPSCs (13). Cells from patients with serious diseases can be reprogrammed back to a pluripotent state and then taken forward again into the cells that were lost during the disease (Fig. 1). Since the first set of disease-specific iPS lines were made (14), there have been many papers showing iPSCs from patients with specific human diseases can reproduce some cardinal features of the disorder (15). In certain cases (especially childhood disorders), the cells recapitulate the damage that was seen in the patients, but now they are in a dish (16). Using iPSC disease-modeling techniques, human diseases can be played over and over again while interrogating real human molecular genetics, disease mechanisms or novel drugs. As if this were not enough, iPSCs may also tell us something about the process of human aging, given that 100-year-old fibroblasts can be reprogrammed back to an embryonic state (17).

This review focuses on some of the latest developments in hiPSC biology, and takes on the heavy task of speculating where this rapidly moving field may be heading over the next few years.

## PROTOCOLS, TOOLS AND TECHNOLOGIES: SHARPENING THE AXE

The power of iPSCs lies in their potential ability to produce any cell in the human body. However, although this is probably possible, there are currently many unresolved issues mainly associated with the maturation of cells to a fully functional state. So rushing into clinical trials before resolving some of these problems may be short-sighted. At a recent bioengineering meeting focusing on using devices and materials to help make iPSCs into miniature organs using synthetic substrates and micro-devices, one of the organizers (William Murphy from the University of Madison, WI, USA) brought up a famous quote from Abraham Lincoln: ‘If I had six hours to chop down the tree, I would spend the first four sharpening the axe’. For iPSCs, sharpening means both improving differentiation protocols to produce functional cells and enhancing gene-editing techniques. This will allow researchers to both produce more appropriate cells for transplantation, and to explore the mechanisms underlying ‘disease in a dish’ models of disease through tagging specific proteins with markers and producing isogenic control lines where possible.

### Neural differentiation leads the way

Interestingly, the most promising cell type that seems to spontaneously arise from iPSCs is neural. This has led to a plethora of publications showing that human iPSCs can make many types of neural cell. Several newer techniques rely on a novel dual smad inhibition step to initiate neural differentiation (18), followed by specific transcription factors and growth factor cocktails to drive the cells toward dopamine neurons (19), motor neurons (20) and striatal neurons (21) to name a few. Curiously, there have been few attempts to make other types of neuronal cells in the brain, such as cerebellar or thalamic neurons. However, methods have been published showing the generation of astrocytes (22) and oligodendrocytes (23) from iPSCs. Furthermore, we recently reported a very simple way to produce a readily expandable neural stem cell that grows as a spherical suspension culture that we termed EZ spheres due to their ease of growth (24). They are capable of making many different neural cell types and were the source of cells for a large collaborative effort to model Huntington's disease using iPSCs (21).

**Figure 3.**
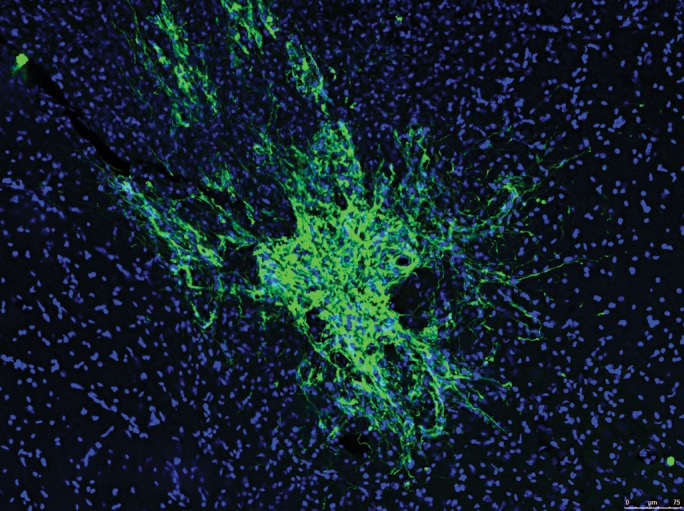
Representative transplant of human iPSC-derived neural cells in the adult rat spinal cord stained with a human cytoplasmic marker (SC121, green) and nuclei stained with Dapi (blue).

Neural cells derived from pluripotent cells can survive and integrate following transplantation into different areas of the rodent central nervous system (Fig. 3). In some cases, these transplants can lead to functional improvements as shown when human ES-derived neural cells were transplanted back into models of Huntington's disease (25) or Parkinson's disease (19) and demyelinating disorders (23). Perhaps the most significant recent advances, though, have been made in the eye, with diseases such as macular degeneration and retinitis pigmentosa caused by deficits in the retinal pigmented epithelium (RPE) at the back of the eye. RPE cells can be easily derived from human pluripotent cells and survive transplantation into animal models of macular degeneration (26,27). These have now been moved into clinical trials for this disorder (28). Japan has also announced that it has approval from their regulatory bodies to take autologous iPSC-derived RPE into the clinic for patients once safety tests have been performed. Clearly, the eye may lead the way for proof of concept that iPSCs can deliver medical therapies, and to test the power of autologous transplantation.

### Others try to follow

Just about every other cell type can also be produced *in vitro* from iPSCs and new protocols constantly arise. Several advanced methods have focused on blood, heart, pancreas, liver and gut but, interestingly, fundamental roadblocks appear to remain for these organ systems. In nearly all cases, differentiation seems to produce immature cells but not mature functional cells required for tissue repair. Perhaps the best example comes from efforts to produce functional blood cells from human iPSCs, where there has been little success in generating a cell type that will engraft into the bone marrow of irradiated mice—one of the features of mature blood cells (29). Another comes from many studies attempting to make functional islet cells from hESCs or hiPSCs as a source of tissue for treating diabetes. Although cells that release insulin in response to glucose can be produced, they appear very frail and do not survive and mature upon transplantation into mice (30). A similar story is found for human iPSC-derived cardiomyocytes that can definitely beat in the culture dish and show some important markers but do not display all of the expected phenotypes of mature cells and survive very poorly following transplantation (31).

Interesting new solutions to these problems are now arising. Simply adding certain reagents such as DMSO to the media may push the cells into a more terminally differentiated state (32). But it also seems like iPSCs may need to be left alone to self-organize into three dimensional cultures *in vitro* or ‘trained’ by an *in vivo* environment before maturing completely. For islet cells and diabetes, the company Viacyte (San Diego, CA) has recently shown that by placing human pluripotent cells differentiating along an islet cell lineage within a capsule, and placing this capsule in a mouse over many months, cells within the capsule start to mature completely and become functional. For the blood system chronically hampered by the inability to achieve an engraftable cell from hiPSC lines, there has been a recent breakthrough. Mice were first injected with teratoma-forming human iPSCs engineered to express green fluorescent protein (GFP). Blood draws from these mice were then transferred to another mouse with irradiated bone marrow which accepted a few of these human GFP-expressing cells that were able to reconstitute the irradiated mouse immune system (33). Together, these studies show that if pluripotent cells are allowed to differentiate over long periods of time in complex 3-D *in vivo* environments, maturation of various cell types can be enhanced (Fig. 1). This provides proof of concept that the cells can mature—we just need to improve differentiation techniques.

Perhaps new ways of differentiating the cells in 3-D substrates will pave the way forward, as shown in exciting new studies where organogenesis from iPSCs has produced structures approximating whole gut (34), liver buds (35) and whole eyes (36). Other ideas relate to growing endothelial cells alongside the maturing cells to stimulate vascular interactions, and using bioengineering to create these complex microenvironments *in vitro*—perhaps through ‘organ on a chip’ technology (37) or cellular printing (38). If the process of full human cell differentiation can be controlled and automated, it opens enormous possibilities, not only for human models of disease but also ultimately as an alternative system for screening and testing the toxicity of human drugs that may lead to faster approval by the Food and Drug Administration (FDA).

### Molecular editing comes of age

One of the most important Nobel Prize-winning molecular techniques that moved the field of animal modeling forward was homologous recombination in mouse ESCs, which allowed editing of their genome and the modern era of transgenics (39). Clearly, editing the genome of iPSCs will also be crucial to move this field forward, as it will allow the knock-out of disease-causing genes and thus production of isogenic, perfectly matched control lines. It will also allow insertion of safety genes, marker genes for specific cell types (via fluorescent proteins behind specific promoters) and inducible genes to switch on and off factors within the differentiating or growing cells.

Early efforts in hESCs relied on the classical homologous recombination techniques and were very inefficient—often taking over a year to target one gene (40). Then zinc finger nucleases (ZFNs) that provided short template complementary sequences combined with integrases were shown to increase the targeting efficiency dramatically and have been successfully applied to both ESCs and iPSCs (41), but are restricted somewhat by high production cost and remaining inefficiency. Combining ZFNs with adenoviral delivery increases efficiency significantly (42), although production of the viral constructs is labor-intensive (43). More recently, transcription activator-like effector nucleases (TALEN) have been used, which are inexpensive to produce, have good specificity and are also very selective, but still the frequency of recombination remains low (44). Finally, CRISPR seems to be very efficient at targeting iPSCs (45) but has shorter recognition arms and thus may suffer from many off-site insertions as shown in a recent publication (46). Human iPSC gene editing is a very quickly evolving area. However, it currently remains the domain of a few selective laboratories or companies due to the highly technical nature of the process and low efficiency (many clones have to be selected and then screened for correct insertion). More efficient ways of gene targeting are desperately needed for use by more laboratories to move the iPSC field forward.

## HUMAN CHIMERIC ANIMAL MODELS OF DISEASE

Transplantation of iPSC-derived cells into animals seems to mature the cells, perhaps because they are now surrounded by a vascular system, immune system and 3-D environment (see section above). So one might envision a new era of disease modeling where human iPSCs are grafted back into immune-compromised mice to form ‘chimeras’. If the iPSCs were derived from a patient with a specific disease caused by a gene mutation or even complex gene interactions, they should mature into organized tissues that may again reflect the disease pathology. These ‘human disease in an animal model’ opens up great opportunities to look at the long-term development of phenotpyes (animal's lifespan) and to test drug therapies in the context of an entire organism. It would also be possible to inject the iPSC-derived cells into humanized mice—immune-deficient mice that are irradiated and injected with human cord blood that takes residence in the bone marrow to give the mouse a human immune system (47). In this model, a number of important questions could be asked. First, it may predict how human cells would react in the context of a human immune system and thus be a good predictor of how immune rejection may occur (see below for more discussion). Second, this may provide the most elegant chimeric model where the human diseased iPSCs differentiate within a mouse with a human immune system and perhaps interact with the immune cells in a way that would accurately predict disease onset and progression.

Clearly, there remain many hurdles to this approach. Mice live only 3 years, which may still not be long enough to produce a relevant phenotype, though their faster aging may provide the human cells with a natural ‘aging accelerator’ required to bring out a phenotype. It is also very labor-intensive to produce humanized mice with individual injections into each animal to create the models, and certain levels of chimerism may raise new ethical concerns. However, if preliminary models prove to substantially enhance disease phenotypes, it may be well worth the investment of time and money to expand these ideas.

## AUTOLOGOUS OR NON-AUTOLOGOUS—THAT IS THE QUESTION

One of the major challenges for whole organ or cellular therapy using stem cells is host immune rejection of the transplanted cells. This is most often overcome by strong immunosuppressive drugs, which have many side effects that may be unavoidable for patients with life-threatening diseases, but not for patients with non-life-threatening diseases that still cause long-term disability. iPSCs in theory provide a promising source of autologous tissue. On the negative side, tumor formation is still a risk and the cells will, of course, carry the gene or predisposition to whatever disease is being treated and may hence behave in the same way following transplantation back into the patient. However, in many cases, the cells in the patient became sick due to a combination of environment, genes and aging; so, ‘rejuvenating’ cells through iPSC generation may give the new source of tissue a new lifespan (see below for expansion of this idea). Furthermore, current gene-editing techniques may allow the mutation to be corrected prior to using the iPSCs, and optimized techniques for fully differentiating and/or sorting iPSCs may remove their risk for tumor formation (see the previous section).

If all this were achieved, these cells, in theory, could be transplanted back into the same patient. But would iPSCs be accepted by the donor? There is the possibility that simply culturing cells might change them enough to stimulate an immune response. A very controversial paper recently suggested this may indeed be the case by showing that mouse autologous iPSCs may be rejected by the same mouse through up-regulation of specific proteins induced by the reprogramming technique (48). However, there have since been a number of recent papers challenging that view and showing that mouse iPSC autologous transplants do not reject (49). Unfortunately, there are many technical issues associated with this area related to the differentiation state of the iPSCs (different cell types may have different stimulatory responses to the immune system) and the transplant region (for example, the brain is known to be immune-privileged). This makes ultimate conclusions difficult to reach. Finally, of course, none of these studies can currently be done with human iPSCs that behave very differently to mouse cells, although new chimeric models of disease may be able to address this issue (see the previous section).

Ultimately, there would be significant advantages to using iPSCs for reducing immune rejection following transplantation. But to move this type of autologous therapy into the clinic requires a number of significant steps. The FDA generally requires each cell product be tested on many animals to assess toxicity and tumorgenicity. This will not be practical for autologous iPSC therapies and will require different guidelines based on standard operating procedures for the generation of cells that are process comparable between different lots. Even if these regulatory hurdles could be overcome, the cost of such a process may be very high. In some cases, this has to be traded against the high cost of immune-suppressing an individual patient over many years. However, for the majority of therapies that would like to use the autologous approach, suppression will not be acceptable due to the high risk of side effects.

Clearly, this is a very complex area. Each field will move forward with different ideas on the use of autologous versus allogeneic iPSC lines taking into account the severity of disease, region of the body being transplanted, economical and regulatory issues and most importantly impact on patient outcome. In some cases, it may be possible to start with an autologous approach to achieve proof of concept that the cell therapy has a clinical effect. Once established, allogeneic lines of cells could then be developed and tested with brief immune suppression in hopes that they would have the same effect. Furthermore, it is also possible to produce banks of iPSC lines representing the major HLA haplotypes which would enable much better matching to occur—perhaps approximating to autologous approaches.

## REVERSING THE AGING PROCESS MAY BE THE LASTING LEGACY OF IPSCs

Although using iPSCs to model human diseases in the dish and as a limitless source of autologous tissue for transplantation are exciting and important, the legacy of iPSCs may be even more dramatic. Producing pluripotent stem cells from adult or even aged fibroblasts feels like getting into a DeLorean and going back in time. However, instead of fiction using a lightning bolt combined with a critical speed of 88 miles per hour to go back in time, it appears that reality suggests all one needs are a few released factors or pluripotency genes which activate endogenous pluripotency pathways. Supporting the possibility of rejuvenation, a number of recent high-profile papers have shown that simply attaching a young mouse to an old mouse can transfer factors from the blood which increase the apparent age of the young mouse while reducing the age of the old mouse (50–52). Using human cells, it has recently been shown that 100-year-old fibroblasts, or younger fibroblasts pushed to senescence in culture, can be reprogrammed to a pluripotent state (17). Remarkably, these cells lose all markers of senescence upon differentiation and behave like those acquired from young individuals. Whether these two ways to decrease apparent age share common mechanisms remains to be established. However, the ability to go back in time is clearly a real possibility through reprogramming. In related studies, adult fibroblasts taken from patients with rapid aging diseases such as Progeria can also be reprogrammed to an embryonic state and look very similar to control iPSCs; however, in contrast to the normally aged fibroblasts, when the Progeria cells were differentiated again, they underwent rapid changes associated with the aging process (53). Thus, if there is a severe genetic mutation causing rapid aging such as in Progeria, this will need to be corrected prior to differentiation; otherwise, the disease will simply be played out again in the dish following differentiation. The Progeria aging phenotype may also be used as an ‘aging stressor’ to elegantly provide the relevance of human aging to models of disease in a dish.

One species has already adapted this reprogramming technique to essentially attain immortality. The jellyfish *Turritopsis nutricula* (cnidaria, hydrozoa) can revert back to an embryonic state when it floats into cooler ocean regions and then back to an adult state in warmer waters through a process of trans-differentiation (54). It remains to be determined whether iPSC approaches induce an ‘all or none’ phenomenon where reprogramming takes cells from old to embryonic with nothing in-between. There will also, of course, be concerns that attempting this *in vivo* will trigger proliferative genes within cells that may create the seeds of cancer. The risks are clearly high. But if modified partial reprogramming could reduce the age of cells *in vivo*, this would be transformational, and potentially lead to rejuvenation in adults that could increase the quality of life and reduce the number of age-related disorders.

## CONCLUSIONS

Regenerative medicine and biology as a whole has been transformed by the ability to reprogram adult cells back to a pluripotent state, which may allow us to cautiously move away from our dependence on immortal human lines and animal models. From disease modeling and organ generation to cellular transplantation and rejuvenation, the possibilities grow rapidly with new high-impact publications. As we understand more about these fascinating cells, manipulate their genomes, place them into bio-matrices and transplant them into living organisms, our knowledge of human disease and potential treatments continues to expand. Perhaps, one day iPSC technology will even begin attacking the challenges of human aging. If nothing else, it has allowed biologists a glimpse into how it might feel to go ‘back to the future’.

## FUNDING

This work was supported by NIH, the Californian Institute for Regenerative Medicine (CIRM) and the ALS Association. Funding to pay the Open Access publication charges for this article was provided by the Cedars-Sinai Regenerative Medicine Institute.

## References

[1] Thomson J.A., Itskovitz-Eldor J., Shapiro S.S., Waknitz M.A., Swiergiel J.J., Marshall V.S., Jones J.M. (1998). Embryonic stem cell lines derived from human blastocysts. Science.

[2] Gurdon J.B. (1962). The developmental capacity of nuclei taken from intestinal epithelium cells of feeding tadpoles. J. Embryol. Exp. Morphol..

[3] Campbell K.H.S., McWhir J., Ritchie W.A., Wilmut I. (1996). Sheep cloned by nuclear transfer from a cultured cell line. Nature.

[4] Takahashi K., Yamanaka S. (2006). Induction of pluripotent stem cells from mouse embryonic and adult fibroblast cultures by defined factors. Cell.

[5] Yu J., Vodyanik M.A., Smuga-Otto K., Antosiewicz-Bourget J., Frane J.L., Tian S., Nie J., Jonsdottir G.A., Ruotti V., Stewart R., Slukvin I.I., Thomson J.A. (2007). Induced pluripotent stem cell lines derived from human somatic cells. Science.

[6] Takahashi K., Tanabe K., Ohnuki M., Narita M., Ichisak T., Tomoda K., Yamanaka S. (2007). Induction of pluripotent stem cells from adult human fibroblasts by defined factors. Cell.

[7] Park I.-H., Zhao R., West J.A., Yabuuchi A., Huo H., Ince T.A., Lerou P.H., Lensch M.W., Daley G.Q. (2008). Reprogramming of human somatic cells to pluripotency with defined factors. Nature.

[8] Sareen D., Svendsen C.N. (2010). Stem cell biologists sure play a mean pinball. Nat. Biotech..

[9] Tachibana M., Amato P., Sparman M., Gutierrez N.M., Tippner-Hedges R., Ma H., Kange E., Fulati A., Lee H.-S., Sritanaudomchai H. (2013). Human embryonic stem cells derived by somatic cell nuclear transfer. Cell.

[10] Yu J., Smuga-Otta K., Tian S., Stewart R., Sluvkin I.I., Thomson J. (2009). Human induced pluripotent stem cells free of vector and transgene sequences. Science.

[11] Zhou H., Wu S., Joo J.Y., Zhou S., Han D.W., Lin T., Trauger S., Bien G., Yao S., Zhu Y. (2009). Generation of induced pluripotent stem cells using recombinant proteins. Cell Stem Cell.

[12] Hou P., Li Y., Zhang X., Liu C., Jingyang G., Li H., Zhao T., Ye J., Yang W., Liu K., Ge J. (2013). Pluripotent stem cells induced from mouse somatic cells by small-molecule compounds. Science.

[13] Merkle F.T., Eggan K. (2013). Modeling human disease with pluripotent stem cells: from genome association to function. Cell Stem Cell.

[14] Park I.-H., Arora N., Huo H., Maherali N., Ahfeldt T., Shimamura A., Lensch M.W., Cowan C., Hochedlinger K., Daley G.Q. (2008). Disease-specific induced pluripotent stem cells. Cell.

[15] Beltrao-Braga P.C., Pignatari G.C., Russo F.B., Fernandes I.R., Muotri A.R. (2013). In-a-dish: induced pluripotent stem cells as a novel model for human diseases. Cytometry A.

[16] Ebert A.D., Yu J., Rose F.F., Mattis V.B., Lorson C.L., Thomson J.A., Svendsen C.N. (2009). Induced pluripotent stem cells from a spinal muscular atrophy patient. Nature.

[17] Lapasset L., Milhavet O., Prieur A., Besnard E., Babled A., Ait-Hamou N., Leschik J., Pellestor F., Ramirez J.M., De Vos J., Lehmann S., Lemaitre J.M. (2011). Rejuvenating senescent and centenarian human cells by reprogramming through the pluripotent state. Genes Dev..

[18] Chambers S.M., Fasano C.A., Papapetrou E.P., Tomishima M., Sadelain M., Studer L. (2009). Highly efficient neural conversion of human ES and iPS cells by dual inhibition of SMAD signaling. Nat. Biotechnol..

[19] Kriks S., Shim J.-W., Piao J., Ganat Y.M., Wakeman D.R., Xie Z., Carrillo-Reid L., Auyeung G., Antoinacci C., Buch A. (2011). Dopamine neurons derived from human ES cells efficiently engraft in animal models of Parkinson's disease. Nature.

[20] Hu B.Y., Zhang S.C. (2009). Differentiation of spinal motor neurons from pluripotent human stem cells. Nat. Protoc..

[21] HD iPSC Consortium (2012). Induced pluripotent stem cells from patients with Huntington's disease show CAG-repeat-expansion-associated phenotypes. Cell Stem Cell.

[22] Krencik R., Weick J.P., Zhang Z.H., Zhang S.C. (2011). Specification of transplantable astroglial subtypes from human pluripotent stem cells. Nat. Biotechnol..

[23] Wang S., Bates J., Li X., Schanz S., Chandler-Militello D., Levine C., Maherali N., Studer L., Hochedlinger K., Windrem M., Goldman S. (2013). Human iPSC-derived oligodendrocyte progenitor cells can myelinate and rescue a mouse model of congenital hypomyelination. Cell Stem Cell.

[24] Ebert A.D., Shelley B.C., Hurely A.M., Onorati M., Castiglioni V., Patitucci T.N., Svendsen S.P., Mattis V.B., McGivern J.V., Schwab A.J. (2013). EZ spheres: a stable and expandable culture system for the generation of pre-rosette multipotent stem cells from human ESCs and iPSCs. Stem Cell Res..

[25] Liu Y., Weick J.P., Liu H., Krencik R., Zhang X., Ma L., Zhou G., Ayala M., Zhang S.C. (2013). Medial ganglionic eminence-like cells derived from human embryonic stem cells correct learning and memory deficits. Nat. Biotechnol..

[26] Lu B., Malcuit C., Wang S., Girman S., Francis P., Lemieux L., Lanza R., Lund R. (2009). Long-term safety and function of RPE from human embryonic stem cells in preclinical models of macular degeneration. Stem Cells.

[27] Zhang K., Liu G.H., Yi F., Montserrat N., Hishida T., Esteban C.R., Belmonte J.C. (2013). Direct conversion of human fibroblasts into retinal pigment epithelium-like cells by defined factors. Protein Cell.

[28] Schwartz S.D., Hubschman J.P., Heilwell G., Franco-Cardenas V., Pan C.K., Ostrick R.M., Mickunas E., Gay R., Klimanskaya I., Lanza R. (2012). Embryonic stem cell trials for macular degeneration: a preliminary report. Lancet.

[29] Ye Z., Chou B.K., Cheng L. (2012). Promise and challenges of human iPSC-based hematologic disease modeling and treatment. Int. J. Hematol..

[30] Nostro M.C., Keller G. (2012). Generation of beta cells from human pluripotent stem cells: potential for regenerative medicine. Semin. Cell Dev. Bio..

[31] Hansson E.M., Lendahl U. (2013). Regenerative medicine for the treatment of heart disease. J. Intern. Med..

[32] Chetty S., Pagliuca F.W., Honore C., Kweudjeu A., Rezania A., Melton D.A. (2013). A simple tool to improve pluripotent stem cell differentiation. Nat. Methods.

[33] Amabile G., Welner R.S., Nombela-Arrieta C., D'Alise A.M., Di Ruscio A., Ebralidze A.K., Kraytsberg Y., Ye M., Kocher O., Neuberg D.S. (2013). In vivo generation of transplantable human hematopoietic cells from induced pluripotent stem cells. Blood.

[34] Spence J.R., Mayhew C.N., Rankin S.A., Kuhar M.F., Vallance J.E., Tolle K., Hoskins E.E., Kalinichenko V.V., Wells S.I., Zorn A.M. (2011). Directed differentiation of human pluripotent stem cells into intestinal tissue *in vitro*. Nature.

[35] Takebe T., Sekine K., Enomura M., Koike H., Kimura M., Ogaeri T., Zhang R.R., Ueno Y., Zheng Y.W., Koike N. (2013). Vacularized and functional human liver from an iPSC-derived organ bud transplant. Nature.

[36] Eiraku M., Takata N., Ishibashi H., Kawada M., Sakakura E., Okuda S., Sekiguchi K., Adachi T., Sasai Y. (2011). Self-organizing optic-cup morphogenesis in three-dimensional culture. Nature.

[37] Huh D., Torisawa Y., Hamilton G.A., Kim H.J., Ingber D.E. (2012). Microengineered physiological biomimicry: organs-on-Chips. Lab Chip.

[38] Tasoglu S., Demirci U. (2013). Bioprinting for stem cell research. Trends Biotechnol..

[39] Mak T.W. (2007). Gene targeting in embryonic stem cells scores a knockout in Stockholm. Cell.

[40] Zwaka T.P., Thomson J.A. (2003). Homologous recombination in human embryonic stem cells. Nat. Biotechnol..

[41] Hockemeyer D., Soldner F., Beard C., Gao Q., Mitalipova M., DeKelver R.C., Katibah G.E., Amora R., Boydston E.A., Zeitler B. (2009). Efficient targeting of expressed and silent genes in human ESCs and iPSCs using zinc-finger nucleases. Nat. Biotechnol..

[42] Liu G.H., Suzuki K., Qu J., Sancho-Martinez I., Yi F., Li M., Kumar S., Nivet E., Kim J., Soligalla R.D. (2011). Targeted gene correction of laminopathy-associated LMNA mutations in patient-specific iPSCs. Cell Stem Cell.

[43] Kim H.W., Svendsen C.N. (2011). Gene editing in stem cells hits the target. Cell Stem Cell.

[44] Ding Q., Lee Y.K., Schaefer E.A., Peters D.T., Veres A., Kim K., Kuperwasser N., Motola D.L., Meissner T.B., Hendriks W.T. (2013). A TALEN genome-editing system for generating human stem cell-based disease models. Cell Stem Cell.

[45] Ding Q., Regan S.N., Xia Y., Oostrom L.A., Cowan C.A., Musunuru K. (2013). Enhanced efficiency of human pluripotent stem cell genome editing through replacing TALENs with CRISPRs. Cell Stem Cell.

[46] Fu Y., Foden J.A., Khayter C., Maeder M.L., Reyon D., Joung J.K., Sander J.D. (2013). High-frequency off-target mutagenesis induced by CRISPR-Cas nucleases in human cells. Nat. Biotechnol.

[47] Legrand N., Ploss A., Balling R., Becker P.D., Borsotti C., Brezillon N., Debarry J., de Jong Y., Deng H., Di Santo J.P. (2009). Humanized mice for modeling human infectious disease: challenges, progress, and outlook. Cell Host Microbe.

[48] Zhao T., Zhang Z.N., Rong Z., Xu Y. (2011). Immunogenicity of induced pluripotent stem cells. Nature.

[49] Guha P., Morgan J.W., Mostoslavsky G., Rodrigues N.P., Boyd A.S. (2013). Lack of immune response to differentiated cells derived from syngeneic induced pluripotent stem cells. Cell Stem Cell.

[50] Villeda S.A., Luo J., Mosher K.I., Zou B., Britschgi M., Bieri G., Stan T.M., Fainberg N., Ding Z., Eggel A. (2011). The ageing systemic milieu negatively regulates neurogenesis and cognitive function. Nature.

[51] Loffredo F.S., Steinhauser M.L., Jay S.M., Gannon J., Pancoast J.R., Yalamanchi P., Sinha M., Dall'Osso C., Khong D., Shadrach J.L. (2013). Growth differentiation factor 11 is a circulating factor that reverses age-related cardiac hypertrophy. Cell.

[52] Ruckh J.M., Zhao J.W., Shadrach J.L., van Wijngaarden P., Rao T.N., Wagers A.J., Franklin R.J. (2012). Rejuvenation of regeneration in the aging central nervous system. Cell Stem Cell.

[53] Liu G.H., Barkho B.Z., Ruiz S.Z., Diep D., Qu J., Yang S.L., Panopoulos A.D., Suzuki K., Kurian L., Walsh C. (2011). Recapitulation of premature ageing with iPSCs from Hutchinson–Gilford Progeria syndrome. Nature.

[54] Piraino S., Boero F., Aeschbach B., Schmid V. (1996). Reversing the life cycle: medusae transforming into polyps and cell transdifferentiation in *Turritopsisnutricula* (cnidaria, hydrozoa). Biol. Bull..

